# Towards a feasible algorithm for tight glycaemic control in critically ill patients: a systematic review of the literature

**DOI:** 10.1186/cc3981

**Published:** 2006-01-26

**Authors:** Sofie Meijering, Anouk M Corstjens, Jaap E Tulleken, John HJM Meertens, Jan G Zijlstra, Jack JM Ligtenberg

**Affiliations:** 1 Medical Doctor, Intensive & Respiratory Care Unit (ICB), University Medical Center Groningen, Groningen, The Netherlands; 2 Anesthesiologist, Intensive & Respiratory Care Unit (ICB), University Medical Center Groningen, Groningen, The Netherlands; 3 Internist-intensivist, Intensive & Respiratory Care Unit (ICB), University Medical Center Groningen, Groningen, The Netherlands; 4 Anesthesiologist-intensivist, Intensive & Respiratory Care Unit (ICB), University Medical Center Groningen, Groningen, The Netherlands; 5 Internist-intensivist, Intensive & Respiratory Care Unit (ICB), University Medical Center Groningen, Groningen, The Netherlands; 6 Internist-intensivist, Intensive & Respiratory Care Unit (ICB), University Medical Center Groningen, Groningen, The Netherlands

## Abstract

**Introduction:**

Tight glycaemic control is an important issue in the management of intensive care unit (ICU) patients. The glycaemic goals described by Van Den Berghe and colleagues in their landmark study of intensive insulin therapy appear difficult to achieve in a real life ICU setting. Most clinicians and nurses are concerned about a potentially increased frequency of severe hypoglycaemic episodes with more stringent glycaemic control. One of the steps we took before we implemented a glucose regulation protocol was to review published trials employing insulin/glucose algorithms in critically ill patients.

**Methods:**

We conducted a search of the PubMed, Embase and Cochrane databases using the following terms: 'glucose', 'insulin', 'protocol', 'algorithm', 'nomogram', 'scheme', 'critically ill' and 'intensive care'. Our search was limited to clinical trials conducted in humans. The aim of the papers selected was required to be glycaemic control in critically ill patients; the blood glucose target was required to be 10 mmol/l or under (or use of a protocol that resulted in a mean blood glucose = 10 mmol/l). The studies were categorized according to patient type, desired range of blood glucose values, method of insulin administration, frequency of blood glucose control, time taken to achieve the desired range for glucose, proportion of patients with glucose in the desired range, mean blood glucose and frequency of hypoglycaemic episodes.

**Results:**

A total of twenty-four reports satisfied our inclusion criteria. Most recent studies (nine) were conducted in an ICU; nine others were conducted in a perioperative setting and six were conducted in patients with acute myocardial infarction or stroke. Studies conducted before 2001 did not include normoglycaemia among their aims, which changed after publication of the study by Van Den Berghe and coworkers in 2001; glycaemic goals became tighter, with a target range between 4 and 8 mmol/l in most studies.

**Conclusion:**

Studies using a dynamic scale protocol combining a tight glucose target and the last two blood glucose values to determine the insulin infusion rate yielded the best results in terms of glycaemic control and reported low frequencies of hypoglycaemic episodes.

## Introduction

Evidence is increasing that tight glycaemic control reduces morbidity and mortality in critically ill patients [1-3]. The study conducted by Van Den Berghe and coworkers in thoracosurgical intensive care unit (ICU) patients [1] yielded impressive results; glycaemic control to a mean blood glucose of 5.7 mmol/l lowered morbidity and mortality by nearly 50%. Following the publication of this trial in 2001, many attempts have been made to achieve strict glycaemic control in ICU patients, with varying and sometimes disappointing results. The glycaemic goals described by Van Den Berghe and coworkers appear difficult to achieve in a real life ICU. Furthermore, most clinicians and nurses are concerned about the potential for an increased frequency of severe hypoglycaemic episodes. Here we review the results of clinical trials using insulin/glucose algorithms in critically ill patients, focusing on the number of blood glucose determinations in the desired range, mean blood glucose and frequency of hypoglycaemic episodes. We provide recommendations for a feasible and reliable insulin/glucose algorithm.

## Materials and methods

We performed a search of the PubMed, Embase and Cochrane databases using the following terms: 'glucose', 'insulin', 'protocol', 'algorithm', 'nomogram', 'scheme', 'critically ill' and 'intensive care'. Our search was limited to full papers of clinical trials in humans. We used the following inclusion criteria: glycaemic control in critically ill patients was the objective of the study; the blood glucose target was 10 mmol/l or under (or the protocol used resulted in a mean blood glucose = 10 mmol/l); and a clear description of the study protocol was given. Studies with patients undergoing only minor surgery were not included. Studies performed with an experimental closed loop, although promising, were also excluded because this system cannot yet be applied in clinical practice. Studies employing glucose-insulin-potassium (GIK) protocols (originally not designed to achieve tight glycaemic control) were included if they satisfied the inclusion criteria.

The abstracts and the abstracts of 'related papers' were evaluated by two researchers (SM and JJML); all papers that satisfied the inclusion criteria were read carefully, and 24 reports were ultimately included in our evaluation. They were categorized according to patient type, desired range of blood glucose values, method of insulin administration, frequency of blood glucose control, time taken to achieve the desired range for blood glucose, proportion patients with blood glucose in the desired range, mean blood glucose and frequency of hypoglycemia. The algorithms used can be divided into whether they use 'sliding' or 'dynamic' scales. With a sliding scale a predetermined amount of insulin is administered, according to the range in which the actual blood glucose value is. For example, every patient with a blood glucose between 5 and 8 mmol/l receives 1 unit of insulin every hour; every patient with a blood glucose between 8 and 11 mmol/l receives 2 units per hour; and so on. In a dynamic scale the dosage of insulin is changed by a certain amount, depending on the range in which the blood glucose is. For example, if blood glucose values are between 6 and 8 mmol/l the actual insulin infusion rate is increased by 1 unit per hour, and if they are between 8 and 10 mmol/l the actual insulin infusion rate is increased by 2 units per hour.

We focused on the results of the group treated using the studied algorithm; the control group was not of interest to the present evaluation.

## Results

### Number of reviewed studies

Twenty-four papers were judged suitable for inclusion because they satisfied the predefined inclusion criteria (Table 1; also see the references list). Most recent studies (nine) were performed in ICUs [1-9]; nine other studies took place in a perioperative setting, mostly in patients with a history of diabetes mellitus [10-18]; and six studies were conducted in patients with acute myocardial infarction or stroke [19-24]. Perioperative studies and studies in myocardial infarction patients were generally of limited duration. Blood glucose targets exhibited wide variation. Before 2001 most studies did not include normoglycaemia among their aims, which changed after publication of the study by Van Den Berghe and coworkers [1]; glycaemic goals became tighter, with a target range between 4 and 8 mmol/l in most studies.

### Methods of insulin administration

Insulin was administered in different ways: subcutaneously, continuous intravenous infusion combined with intravenous bolus injections, or insulin combined with glucose and potassium (glucose-insulin-potassium [GIK] infusion).

#### Subcutaneous insulin

Three studies employed subcutaneous insulin injections. In a limited study conducted in perioperative diabetic patients [15] the target range (5–10 mmol/l) was achieved in only 40% of patients. In another limited study (19 patients) [10], reasonable control was achieved during a 48-hour postoperative period (mean glucose 7.2 ± 1.2 mmol/l). Krinsley [2] administered subcutaneous insulin to 800 mixed ICU patients but switched the route of administration to intravenous in the event of failure to achieve glycaemic control; a blood glucose level below 7.7 mmol/l was achieved in 69% of patients. In conclusion, only a few studies employed subcutaneous insulin therapy alone. Subcutaneous therapy, followed by intravenous insulin if needed, resulted in glycaemic control in only two-thirds of ICU patients.

#### Continuous insulin infusion

Most study protocols used continuous intravenous insulin infusion combined with intravenous bolus injections.

#### Sliding scale protocols

Most studies using a sliding scale protocol resulted in moderate to disappointing regulation of blood glucose, despite blood glucose measurements every 1–4 hours. In a study conducted in diabetic patients undergoing cardiac surgery [12] the mean blood glucose was 9.5 mmol/l; in another study conducted in 29 diabetic patients with acute myocardial infarction [22] the level was 8.2 mmol/l; and in a third study conducted in 35 diabetic patients with acute myocardial infarction [24] the level was 10.3 mmol/l compared with a target of 4–8 mmol/l.

#### GIK protocols

An alternative way to administer insulin is in one solution with glucose and potassium (for example, GIK infusion; also known as GIPS). GIK protocols were originally not designed to acheive tight glucose regulation; this might explain why the results of GIK are variable in terms of glycaemic control.

In a study conducted in diabetic patients with acute medical diseases [9] the target of 6–8 mmol/l was not achieved despite hourly blood glucose measurements (mean blood glucose 10.1 mmol/l). In a recent study employing short-term GIK infusion and additional bolus insulin injections in patients undergoing cardiac surgery [11], a mean blood glucose of 7.7 ± 0.2 mmol/l was reached. In a GIK study conducted in acute stroke patients [21], 24% of patients had BG values above the target range of 4–7 mmol/l during the first 24 hours.

#### Dynamic scale protocols

In critically ill patients the best results are attained in studies using a dynamic scale protocol. The most tight glycaemic control (normoglycaemia) was achieved by Van den Berghe and coworkers [1] in 765 thoracosurgical patients; the mean morning blood glucose was 5.7 mmol/l. Hypoglycaemia (<2.2 mmol//) was identified in 5% of patients. Recently, in a study conducted in a mixed medical-surgical ICU population [3], blood glucose levels were between 4.5 and 6.1 mmol/l for 11.5 hours per day, with a reduction in the incidence of severe hypoglycaemia from 16% to 4% after implementation of the study protocol. Unfortunately, the report provides no information regarding the mean blood glucose. In a recent study conducted in 27 mixed ICU patients [4] a median blood glucose of 6.6 mmol/l was reported. Dazzi and coworkers [5] performed a study in 20 critically ill diabetic patients; the mean blood glucose was 7.8 mmol/l. The frequency of hypoglycaemia was low, with no blood glucose values below 2.5 mmol/l.

In general, these recent studies using a dynamic scale yielded better results in terms of glycaemic control to predefined targets and a low frequency of hypoglycaemic episodes compared with studies using sliding scale protocols. They all combine a tight glucose target and the use of the last two blood glucose values to determine the insulin infusion rate [1,3-7]].

### Methods of blood glucose determination

Most studies used handheld meters with strips for blood glucose determination at the bedside. In the study by Van Den Berghe and coworkers [1], an ICU-based blood gas/blood glucose analyzer was used. In the evaluated studies blood glucose was measured in arterial, venous, or capillary blood samples.

## Discussion

Tight blood glucose control in critically ill patients can best be achieved using a protocol with continuous insulin infusion combined with frequent blood glucose determinations and the use of the last two blood glucose values to determine the insulin infusion rate. Although there is much concern about hypoglycaemia, the frequency of severe hypoglycaemic episodes has been found to be less than 4–5%; in some studies this was even lower than with protocols used in the control groups.

Debate is ongoing regarding the desired BG target. In the real life ICU any change in blood glucose level toward the normal range with an insulin infusion protocol will probably improve hospital survival and reduce morbidity both in surgical and in medical ICU patients [1,2,25]. The mechanisms underlying the effects of glucose toxicity or the possibility of beneficial effects of insulin infusion *per se *remain to be unravelled [26,27]. At present there is no strong evidence that regulation between 4 and 6 mmol/l is more beneficial then regulation between 6 and 8 mmol/l. Most studies apparently aimed for a somewhat higher, probably more feasible, target. On the other hand, ongoing trials such as the Portland protocol [28] have set lower target ranges – between 4.4 and 6.6 mmol/l.

The advice given in the recent Surviving Sepsis Campaign Guidelines [29] – specifically to maintain blood glucose level below 8.3 mmol/l following initial stabilization – seems practical and safe in common clinical practice, but will not always result in improvement of glucose regulation in every ICU. For our medical ICU we calculated a mean blood glucose of all patients admitted in 2000–2001 of 7.5 ± 2.9 mmol/l, which was achieved with insulin administration prescribed at the physician's discretion [30]. To achieve an improvement in morbidity and mortality, we must probably select a blood glucose target lower than 7.5 mmol/l.

In most studies handheld meters with strips were used. The literature on point-of-care testing suggests that accuracy varies with the different handheld meters. Because we found that an ICU-based blood gas/blood glucose analyzer had the best correlation coefficient with our gold standard (central clinical laboratory measurement), we prefer using this device to handheld meters [28]. Furthermore, the studies evaluated here used capillary, venous, or arterial blood for glucose determination. It is known that full blood glucose and plasma glucose values differ, and the same is true for arterial and venous blood samples.

In summary, we can make the following recommendations regarding the implementation of a feasible glucose regulation protocol. First, choose a blood glucose target between 4 and 8 mmol/l. How 'low' depends on local possibilities (personnel, workload, fast and accurate point-of-care blood glucose determination, among other factors) and on the prevailing mean blood glucose level before starting a protocol. Second, it is preferable to use a dynamic scale protocol with continuous insulin infusion combined with frequent blood glucose determinations (hourly to every four hours) and to use the last two blood glucose values to determine the insulin infusion rate. Feasible protocols can be found in the recent literature (Table 1; also see the references list). Third, tight regulation without hypoglycaemia is probably easier to achieve if continuous enteral feeding can be provided. Whether continuous glucose infusion is necessary before enteral feeding is started is not clear yet. Finally, frequent blood glucose determinations impose increased nursing workload, and acceptance of the protocol by nurses is very important for successful implementation. Training, education and subsequently feedback is necessary to motivate ICU nurses [3,7].

## Conclusion

Tight glycaemic control in critically ill patients can best be achieved using a protocol involving continuous insulin infusion combined with frequent blood glucose determinations (hourly to 4 hourly) and the use of the last two blood glucose values to determine the insulin infusion rate. The blood glucose target to aim for must be between 4 and 8 mmol/l and depends on local possiblities (personnel, fast and accurate point-of-care blood glucose determination, among other factors) and on the prevailing mean blood glucose level before starting a protocol. Acceptance of the protocol by nurses is very important for successful implementation.

## Key messages

• 	Tight glycaemic control in critically ill patients can best be achieved using a protocol with continuous insulin infusion combined with frequent blood glucose determinations and the use of the last two blood glucose values to determine the insulin infusion rate.

• 	The blood glucose target must be between 4 and 8 mmol/l and depends on local possibilities (personnel, fast and accurate point-of-care blood glucose determination, among other factors) and on the prevailing mean blood glucose level before starting a protocol.

• 	Frequency of severe hypoglycaemia may even be lower than with existing 'routine' protocols.

• 	Acceptance of the protocol by nurses is important for successful implementation.

## Abbreviations

GIK = glucose-insulin-potassium, ICU = intensive care unit.

## Competing interests

The authors declare that they have no competing interests.

## Authors' contributions

SM and JJML conducted the study, collected data, and drafted the manuscript. AMC, JGZ, JET and JHJMM assisted in writing the manuscript. All authors read and approved the final manuscript.

## Figures and Tables

**Table 1:** Summary of studies included in the present evaluation

Ref. (year)	Number of patients and category	Blood glucose target (mmol/l)	Method	Used BG meter	Frequency of measurements	Hypoglycaemia (mmol/l)	Results (mmol/l)
[1]	765 Thoracosurgical ICU patients	4.4–6.1	Start at BG >6.1 mmol/l. Insulin ± 0.1–2 IU/hour depending on last two BG values. Glucose infusion or feeding	ICU based ABL700^® ^bloodgas/BG analyzer. Arterial blood samples	1–4 hours	5.1% of patients <2.2	Mean morning BG 5.7 ± 1.1
[4] (2004)	27 Mixed ICU patients	4–7	Bath IIP: insulin ± 0.5–4 IU/hour, depending on last two BG values	Accu Check^® ^Advantage 2. Mostly arterial samples	1–2 hours	Three BG values <2.2	Median BG 6.6
[2] (2004)	800 Mixed ICU patients	<7.7	Insulin sc. If two BG values >11.1 mmol/l: insulin iv, sliding scale. Glucose infusion/feeding	Finger stick samples or plasma BG (Vitros^® ^lab analyzer)	Every 3 hours if sc, hourly if iv	0.34% of patients <2.2	Mean BG 7.2 70% of BG <7.7
[5] (2001)	20 Critically ill diabetic patients	6.7	Insulin iv between -1.5 and +1.5 IU/hours depending on last two BG values. Glucose-potassium infusions	OneTouch^® ^II. Capillary samples	4 hours	No BG <2.5	Mean BG 7.8 ± 0.2
[6] (2004)	118 Cardiothoracic ICU patients	5.5–7.7	Yale IIP. Insulin ± 0.5–10 IU, depending on last two BG values and infusion rate	OneTouch^® ^Surestep Flex	1 hour	0.2% of BG <3.3	73% between 4.4 and 7.7
[7] (2004)	52 Medical ICU patients	5.5–7.7	Yale IIP. Insulin ± 0.5–10 IU, depending on last two BG values and infusion rate	OneTouch^® ^Surestep Flex	1–4 hours	0.3% of BG <3.3	66% between 4.4 and 7.7
[3] (2004)	50 Mixed ICU patients	4.5–6.1	Insulin ± 0.5–2 IU/hour, depending on last two BG values. Dextrose infusion or feeding	Accu Check^® ^Inform. Capillary samples	1–2 hours	4% of patients <2.2	11.5 ± 3.7 hours/day between 4.5 and 6.1
[8] (2004)	168 Cardiothoracic ICU patients	4.4–8.3	Insulin 1–16 IU/hour + bolus, sliding scale, depending on last BG value	Accu Check^® ^Inform. Venous samples	1–4 hours	7.1% of BG <2.2	61% of BG between 4.4 and 8.3
[9] (2003)	17 Diabetic patients, acute medical diseases	6–7	GIK + bolus, insulin 1–4 IU/h, dynamic scale, depending on last BG value	Hemocue^® ^meter. Capillary samples	1 hour	Four times	Mean BG 10.1
[10] (2002)	37 Postsurgical NIDDM patients		19 patients sc, 18 patients iv, sliding scale, 5% glucose infusion	Glucometer^®^. Capillary samples	4 hours	5.6% of patients in iv group	sc mean: 7.2 ± 1.2 iv mean: 7.3± 1.1
[11] (2004)	72 Cardiothoracic diabetic patients	6.9–11.1	GIK protocol. Continuous GIK infusion + insulin bolus if BG >15 mmol/l	?	1 hour		Mean BG 7.7 ± 0.2
[31] (1996)	60 Surgical NIDDM patients	3.3–11.1	Insulin bolus if BG >11.1 mmol/l. Group 1: saline. Group 2: glucose-insulin. Group 3: bolus every 2 hours	Capillary samples	15 min	No	Mean BG <9 within all groups
[12] (2002)	29 Diabetic patients, cardiac surgery, 5 days	6.7–11.1	Start if BG >7.8 mol/l. Sliding scale	Venous and capillary samples	Six measurements per day	0.2% of BG <3.8	Mean BG 9.5
[13] (1997)	595 Diabetic patients, cardiac surgery	<11.1 l	Portland protocol: insulin depending on last two BG values and insulin infusion rate	Glucometer	1–2 hourly		Mean BG 9.7
[14] (1987)	24 Diabetic patients after surgery	6.7–10	Insulin ± 0.5 IU/hour depending on BG. Bolus if BG >13.3 mol/l. 5% dextrose infusion	Accu Check + strips	2 hourly	1.4% of measurements	Mean BG between 6.7 and 10
[15] (1988)	30 Diabetic patients, perioperative	5–10	Group 1: iv, every 4 hours ± 0.5 IU/hour. Group 2: sc, every 4 hours ± 2 IU/4 hours. Glucose-potassium infusion	Glucometer	Hourly during surgery, 4 hourly after surgery	0.6% of measurements <2.8 in iv group	67% of iv group between 5.0 and 10; 40% in sc group
[17] (2002)	188 Patients, during cardiac surgery	4.4–6.6	Start (2 IU/hour) with BG >6.6 mmol/l. Double infusion rate until BG <6.6 mol/l.	?	20 min	12% of patients with BG <3.8	In 23% of patients BG <8.3
[18] (1994)	77 Diabetic patients, surgery	6.7–10	Insulin ± 0.5–1.0 U/hr depending on BG	Reflolux S (+strips) and Glucose hexokinase (lab)	4 hourly, hourly during surgery	Two patients	62% of patients BG between 3.5 and 15.0
[19] (2000)	24 Type 2 diabetic patients, acute myocardial infarction	8.3–11.0	Insulin ± 1–2 IU/h, depending on BG range.	Venous samples. Automatic analyzer (lab)	30 min to 2 hours		Mean BG 6.9 ± 0.8
[20] (2002)	25 Diabetic patients, acute coronary syndromes	6.6–8.2	Insulin change by -1 to +3 IU/hour, depending on BG	Beckman^® ^glucose analyzer II	1–3 hourly	Four patients with mild hypoglycaemia	Mean 7.2 ± 1.7
[21] (1999)	25 Patients, acute stroke (during 24 hours)	4–7	GIK: 500 ml glucose 10% + 16 U insulin + 20 mmol KCl; 100 ml/hour. ± 4 U insulin/infusate, depending on BG	BM Glycemic strips	2 hourly	One patient	Mean BG of 68% of patients <7
[22] (1992)	29 Diabetic patients, acute myocardial infarction	4–8, to reach within 4 hours	Sliding scale, more insulin with left ventricular failure and bodyweight >120 % of ideal	Capillary samples	1–4 hourly	1.2% of BG <3	Mean BG 8.2 ± 1.3
[23] (1994)	158 Diabetic patients, acute myocardial infarction	7–10	>15 mmol/l; bolus iv. Insulin ± 0.5–1 IU/hour depending on BG. Glucose infusion	Venous samples. Reflolux II	1–2 hourly	17.7% of patients with BG <3.0 mmol/l	Mean BG 9.2 ± 2.9 after 24 hours
[24] (1991)	35 Diabetic patients, acute myocardial infarction	4–8	Sliding scale. Dextrose 5% infusion	Glucometer II^®^. Capillary samples	2–4 hourly		Mean BG 10.3 ± 2.1

Studies mentioned in the table are arranged according to patient category. Intensive care patients at the top, followed by surgical patients, divided in patients undergoing general surgery and patients undergoing cardiac surgery. The third category of patients consists of patients with an acute myocardial infarction. BG, blood glucose; IIP, insulin infusion protocol; iv, intravenous; NIDDM, noninsulin-dependent diabetes mellitus; sc, subcutaneous.
